# Anlotinib for Patients With Metastatic Renal Cell Carcinoma Previously Treated With One Vascular Endothelial Growth Factor Receptor-Tyrosine Kinase Inhibitor: A Phase 2 Trial

**DOI:** 10.3389/fonc.2020.00664

**Published:** 2020-05-07

**Authors:** Jianhui Ma, Yan Song, Jianzhong Shou, Yuxian Bai, Hanzhong Li, Xiaodong Xie, Hong Luo, Xiubao Ren, Jiyan Liu, Dingwei Ye, Xianzhong Bai, Cheng Fu, Shukui Qin, Jinwan Wang, Ai-Ping Zhou

**Affiliations:** ^1^Department of Urology, National Cancer Center/National Clinical Research Center for Cancer/Cancer Hospital, Chinese Academy of Medical Sciences and Peking Union Medical College, Beijing, China; ^2^Department of Medical Oncology, National Cancer Center/National Clinical Research Center for Cancer/Cancer Hospital, Chinese Academy of Medical Sciences and Peking Union Medical College, Beijing, China; ^3^Department of Gastrointestinal Oncology, Harbin Medical University Cancer Hospital, Harbin, China; ^4^Department of Urology, Peking Union Medical College Hospital, Beijing, China; ^5^Department of Oncology, General Hospital of Northern Theater Command, Shenyang, China; ^6^Department of Urology and Oncology, Chongqing University Cancer Hospital, Chongqing Cancer Hospital, Chongqing, China; ^7^Department of Biotherapy, Tianjin Medical University Cancer Institute and Hospital, National Clinical Research Center for Cancer/Key Laboratory of Cancer Prevention and Therapy, Tianjin/Tianjin's Clinical Research Center for Cancer, Tianjin, China; ^8^Department of Biotherapy, Cancer Center, West China Hospital, Sichuan University, Chengdu, China; ^9^Department of Urology, Fudan University Shanghai Cancer Center, Shanghai, China; ^10^Department of Urinary Surgery, Guangxi Medical University Affiliated Tumor Hospital, Nanning, China; ^11^Department of Urology, Cancer Hospital of China Medical University, Liaoning Cancer Hospital & Institute, Shenyang, China; ^12^Cancer Center, Jinling Hospital, Nanjing, China

**Keywords:** anlotinib, metastatic renal cell carcinoma, tyrosine kinase inhibitor, second-line, FGFR

## Abstract

**Introduction:** Sequential therapy with vascular endothelial growth factor receptor-tyrosine kinase inhibitors (VEGFR-TKIs) is effective in some patients with metastatic renal cell carcinoma (mRCC) progressed from or were intolerant to a prior TKIs. Anlotinib is a multi-kinase inhibitor targeting VEGFR1/2/3, PDGFR and FGFR, which has demonstrated efficacy and safety in first-line treatment of mRCC. This study assessed the potential of anloitnib as second-line treatment for patients with mRCC after prior one VEGFR-TKI.

**Methods:** This is a single-arm, open-label, phase 2 study. Patients progressed after or were intolerant to sorafenib or sunitinib were enrolled. Anlotinib was administrated orally 12 mg once daily for 14 days every 3 weeks. The primary endpoint was progression-free survival (PFS). Secondary endpoints included overall survival (OS), objective response rate (ORR), safety and quality of life (QoL).

**Results:** Forty three patients were enrolled and 42 received anlotinib, of whom 32 progressed after and 10 were intolerant to sorafenib or sunitinib. Median PFS were 14.0 months (95% CI 8.3–20.3) and 8.5 months (95% CI 5.6–16.6) for overall population and patients progressed after a previous VEGFR-TKI, respectively. Median OS was 21.4 months (95% CI 16.0–34.5), confirmed ORR and DCR were 16.7 and 83.3% in overall population. The most common adverse events included diarrhea (47.6%), hypertension (45.2%), hand and foot syndrome (42.9%), and fatigue (40.5%). Grade 3 hematological adverse events occurred in four cases, while no grade 4 hematological adverse events was observed.

**Conclusions:** Anlotinib showed promising efficacy as well as favorable safety as second-line treatment for patients with mRCC.

**Clinical Trial Registration:** www.ClinicalTrials.gov, identifier: NCT02072044.

## Introduction

Renal cell carcinoma (RCC) is highly vascularized (1). Tyrosine kinase inhibitors (TKIs) that target the vascular endothelial growth factor receptor (VEGFR) have become the backbone in the first-line treatment for patients with metastatic renal cell carcinoma (mRCC) for more than 10 years (2), and the survival for VEGFR-TKI monotherapy was ~28.4–31.5 months compared with 13 months in the era of cytokines (3–6). With the development of immunotherapy, progression-free survival has been further improved with combination regimens such as nivolumab/ipilimumab, avelumab/axitinib, and pembrolizumab/axitinib, which have been new choices for the first-line and second-line therapy while long-term survival still need confirmation (7–9). Seven TKIs and monoclonal antibodies targeting angiogenesis have been approved, including sunitinib, pazopanib, axitinib, cabozantinib, and tivozanib recently. However, most patients will progress from first-line anti-angiogenic therapy after a PFS of 8.4–11 months (3, 4, 10) and are in need of second-line therapy including sequential another VEGFR-TKIs (e.g., cabozantinib or axitinib), mTOR inhibitors or the combination therapy. Besides, the approval of nivolumab for mRCC in Nov 2015 indicated that immunotherapy had become another option in the second-line therapy (11).

Although the choices of second-line therapy for mRCC has been extended, durable anti-angiogenic therapy will continue to be one of the most important strategies (12). Besides VEGFR as the primary target (13), several anti-angiogenic TKIs including cabozantinib and lenvatinib have additional targets related to the resistance of anti-angiogenesis such as fibroblast growth factor receptor (FGFR), AXL and MET (14, 15). The activation of FGFR pathway engages the escape of anti-VEGF/VEGFR therapy (16). Therefore, agents with inhibitory activity on FGFR may be more effective after the failure of first-line VEGFR TKI and have been investigated in clinical trials (17).

Anlotinib is an oral multikinase inhibitor blocking VEGFR, FGFR, platelet-derived growth factor receptors α/β (PDGFRα/β) and c-Kit (18). *In vitro* studies, anlotinib selectively inhibited VEGFR2 with an IC_50_ value of 0.2 nM as 20-fold higher inhibitory activity than sunitinib (19). Anlotinib inhibits the activation of FGFR by blocking the phosphorylation of FGFR1 on an inhibition rate of 45.0% (p-FGFR1/FGFR1) at 1 μM, and showed an IC_50_ value of 25 nM in AN3Ca cells overexpressing a FGFR2 mutant protein in another assay (20, 21). Anlotinib at the dose of 12 mg on a 2/1 schedule has displayed favorable tolerance as well as lasting and broad-spectrum antitumor activity in a phase 1 trial in which 2/4 patients with mRCC achieved PR (22). In China, anlotinib has been approved for the third-line treatment for non-small cell lung cancer and second-line treatment for soft tissue sarcoma (23, 24). For the strong inhibitory activity against VEGFR2 and FGFR, as well as the favorable safety profile, we interpreted a randomized phase 2 study to compare the efficacy of anlotinib and sunitinib as first-line therapy for mRCC (ClinicalTrial.gov, number NCT02072031) and demonstrated similar efficacy and better safety profile of anlotinib compared with sunitinib (25). At the same time, we launched a single-arm phase 2 study to investigate the efficacy and safety of anlotinib in patients with mRCC after first-line anti-angiogenic TKI treatment (ClinicalTrial.gov, number NCT02072044). Here we report the final results of this study.

## Materials and Methods

### Study Design

This is a prospective, multicenter, single-arm study involved 11 hospitals in China. The study was approved by the institutional ethics committees, following the principles of Declaration of Helsinki and Good Clinical Practice promulgated by National Medical Products Administration of China. Written consents were obtained from all patients with thorough explanation of the potential risks and benefits of the protocols. Anlotinib was provided by Chia Tai TianQing Pharmaceutical Group Co., Ltd. (China).

### Patients

Eligible patients were 18–75 years of age, diagnosed with measurable, unresectable and histologically confirmed mRCC with a clear cell component. All patients had progression disease after or were intolerant to previous sorafenib or sunitinib. Patients were required for an Eastern Cooperative Oncology Group performance status (ECOG PS) of 0–1 and adequate organ function, based on standard laboratory tests including hematology, serum chemistry, coagulation, thyroid function, left ventricular ejection fraction and urinalysis.

The main exclusion criteria included: uncontrolled blood pressure (systolic pressure > 140 mmHg or diastolic pressure > 90 mmHg with adequate anti-hypertension medication), active myocardial ischemia, history of arterial infarction, QT interval ≥440 millisecond (ms) or cardiac insufficiency, 24 h urine protein >1.0 g; venous thrombosis within 6 months; clinically significant hepatic or gastrointestinal dysfunction, wound healing and infectious comorbidities.

### Drug Administration

All patients received oral anlotinib hydrochloride capsules once daily at a dose of 12 mg on day 1–14, every 3 weeks (2/1 schedule). Treatment was continued until disease progression or intolerable toxicity. Dose reduction to 10 mg per day was allowed when grade 3 non-hematology or grade 4 hematology adverse events occurred. A minimum dose of 8 mg was allowed when the adverse events occurred again.

### Endpoints

The primary endpoint was progression-free survival (PFS), defined as the time from first date of drug administration to the time of disease progression according to RECIST version 1.1 or death for any reason. Secondary endpoints included overall survival (OS), objective response rate (ORR), disease control rate (DCR), and safety. OS was measured from the first date of drug administration to the date of death for any reason with follow-up every 3 months. Patients who were event-free or lost to follow-up were censored at the time of last visit. ORR was the sum of complete response (CR) and partial response (PR). DCR refers to the proportion of patients with CR, PR, and stable disease (SD) lasting for 12 weeks or more. Tumor response was assessed according to Response Evaluation Criteria in Solid Tumors (RECIST) version 1.1. Functional Assessment of Cancer Therapy-Kidney Symptom Index-15 (FKSI-15) (26) and Functional Assessment of Cancer Therapy-Kidney Cancer Symptom Index-disease-related symptoms subscale (FKSI-DRS) (27) were used to evaluate the disease related symptoms and quality of lofe (QoL).

### Statistical Analysis

In AXIS study for axitinib, median PFS for patients with advanced RCC after sunitinib was 4.8 months (28). Considering patients who were intolerant to previous TKIs were included, a relative longer PFS should be achieved in this study. Thus, our study was designed to detect an improvement in median PFS from 4.8 to 8.3 months, corresponding to a 42% decrease in HR. A total of 33 patients were required based on 80% power and a two-sided test at a significance level of 0.05, and 42 patients were planned for enrollment.

## Results

### Study Population

**Figure 1:**
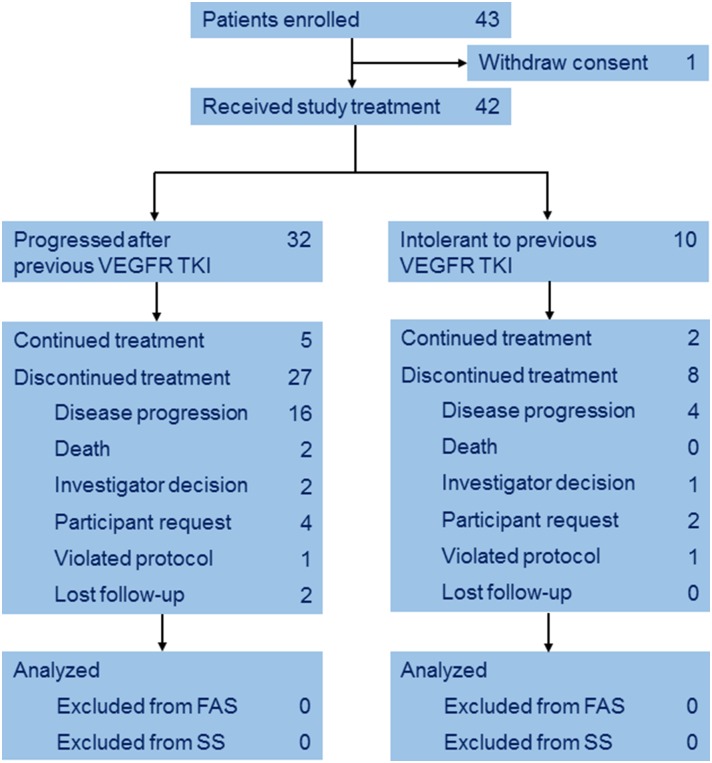
CONSORT diagram.

Between Mar 2014 and Mar 2015, a total of 43 eligible patients with mRCC were enrolled from 11 institutions. One patient withdrew consent before treatment (Figure 1). Baseline characteristics of the 42 subjects are listed in Table 1. The median age was 59 years (IQR 53–62), 10 (23.8%) patients were intolerant to and 32 (76.2%) progressed after previous TKI. 23 (54.8%) patients received previous sunitinib and the others received pervious sorafenib.

**Table 1:** Patient clinical and demographic data.

	**Patients *N* (%)**
**Sex**, ***n***	
Male	31 (73.8)
Female	11 (26.2)
**Age (years)**	
Median	59.0
Range	29-74
**Previous therapies**	
Surgery	36 (85.7)
Chemotherapy	7 (16.7)
Radiotherapy	6 (14.3)
Sunitinib or Sorafenib failure	32 (76.2)
Sunitinib or Sorafenib intolerant	10 (23.8)
Other antitumor therapies	17 (40.5)
**ECOG PS**	
0	9 (21.4)
1	33 (78.6)
**Metastatic site**	
Lung	28 (66.7)
Liver	4 (9.5)
Bone	14 (33.3)
Lymph node	9 (21.4)
Others	11 (26.2)

### Treatment

Median duration of treatment was 8.3 months (IQR 4.2–16.4). Twenty eight patients received more than 8 cycles, while one and six patients received 7 and 6 cycles, respectively. Dose reductions were reported in five (11.9%) patients.

### Efficacy

**Figure 2:**
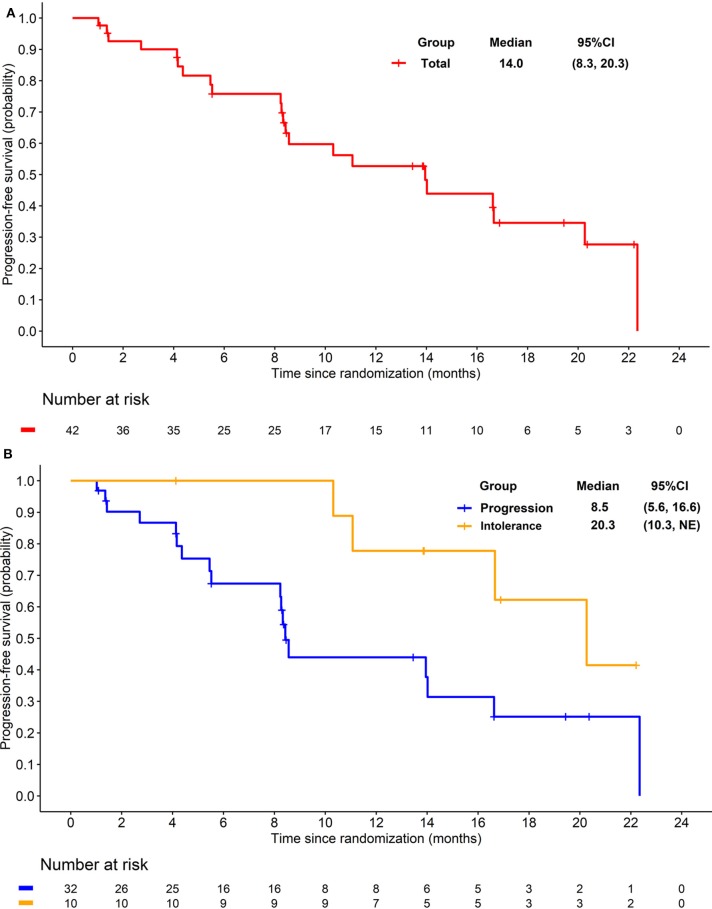
**(A)** Kaplan-Meier plot of progression free survival in overall patients. **(B)** Kaplan-Meier plot of progression free survival in patients progressed from previous TKI treatment (Blue) and intolerant to previous TKI treatment (yellow).

**Figure 3:**
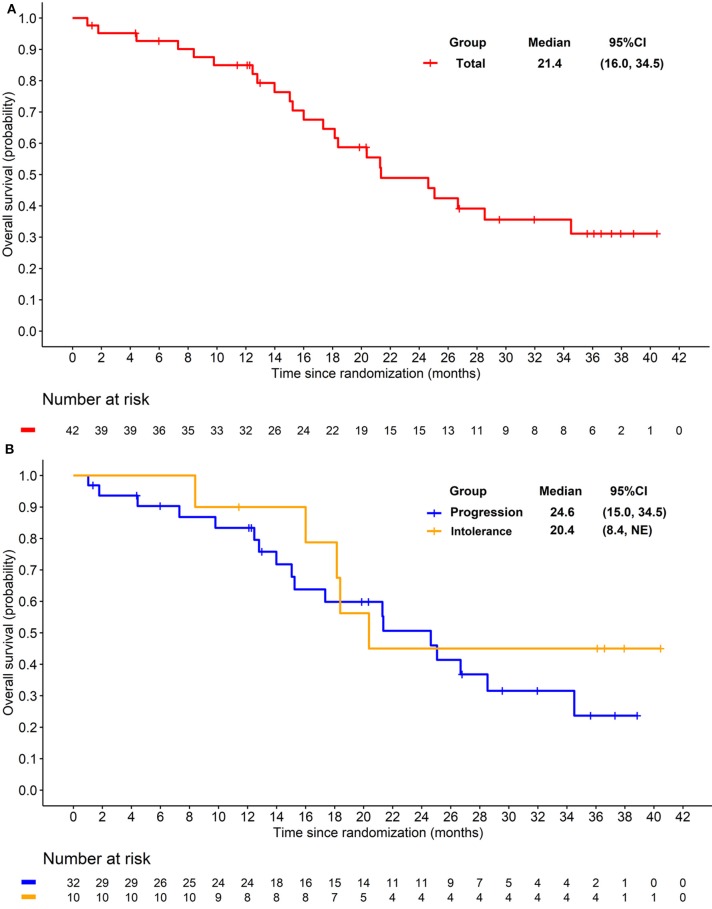
**(A)** Kaplan-Meier plot of overall survival in overall patients. **(B)** Kaplan-Meier plot of overall survival in patients progressed from previous TKI treatment (Blue) and intolerant to previous TKI treatment (yellow).

The preliminary analysis was conducted on May 2015 and the median PFS (mPFS) was 11.8 months, which has been reported at the 2016 ASCO annual meeting (29). After that, treatment was continued for patients without PD or death. On the cut-off date of Apr 25, 2016 for primary endpoint, the median PFS (mPFS) of 14.0 months (95% CI 8.3–20.3; Figure 2A) was achieved. For the 32 patients progressed after previous TKI, mPFS was 8.5 months (95% CI 5.6–16.6; Figure 2B), while for the 10 patients who were intolerant to previous TKI, mPFS was 20.3 months (95% CI 10.3-NE; Figure 2B). After the cut-off date for PFS, follow-up was continued for 16 months and the median duration of follow-up for OS was 18·3 months (IQR 12·1–28.5). Median OS were 21.4 months (95% CI 16.0–34.5; Figure 3A) with the whole population, 24.6 months (95% CI 15.0–34.5; Figure 3B) and 20.4 months (95% CI 8.4-NE; Figure 3B) with the two groups, respectively. In subgroup analysis for patients previously treated with sorafenib or sunitinib, respectively, mPFS were 20.3 months (95% CI 8.3–22.3) and 14.0 months (95% CI 5.5–16.7), OS were 25.1 (95% CI 15.0-NE) months and 21.3 (14.0-NE) months.

There was no CR observed in the study. Eight patients achieved PR and 30 had SD in the initial evaluation. At final analysis, seven (16.7%) achieved confirmed objective response and 28 (66.7%) had stable disease. Therefore, 35 (83.3%) patients achieved disease control. For patients progressed after previous TKI, ORR and DCR were 15.6 and 78.1%, for patients who were intolerant to previous TKI, ORR, and DCR were 20.0 and 100% respectively.

Kidney-relevant symptoms and functions assessed by FKSI-15 and FKSI-DRS scoring are summarized in Table 2. Compared to the baseline, no significant difference was observed in scores of FKSI-15 or FKSI-DRS after 8 cycles of study treatment.

**Table 2:** Assessment of the quality of life after anlotinib treatment by FKSI-15 and FKSI-DRS scoring.

**Treatment**	***n***	**Assessment**	**Mean ± SD**	**Median**	**Range**	***p***
Baseline	41	FKSI-15	49.41 ± 9.28	53	23–60	–
		FKSI-DRS	30.56 ± 5.08	32	17–36	–
Cycle 2	38	FKSI-15	50.53 ± 7.78	51.5	27–60	0.93
		FKSI-DRS	30.95 ± 4.81	31.5	15–36	0.97
Cycle 4	34	FKSI-15	49.15 ± 7.99	49.5	25–60	0.07
		FKSI-DRS	30.47 ± 4.42	31	16–36	0.18
Cycle 6	31	FKSI-15	49.48 ± 9.10	52	24–60	0.40
		FKSI-DRS	30.19 ± 5.50	32	13–36	0.26
Cycle 8	22	FKSI-15	52.14 ± 6.94	54	34–59	0.94
		FKSI-DRS	32.27 ± 3.77	33	22–36	0.87

### Safety

Treatment with anlotinib was well tolerant. Adverse events occurred in 88.1% patients (Table 3, Supplementary Table 1) and most were grade 1 or 2. The most common adverse events (>20%) were diarrhea (47.6%), hypertension (45.2%), hand and foot syndrome (42.9%), fatigue (40.5%), proteinuria (35.7%), anorexia (33.3%), hypothyroidism (28.6%), hoarse voice (26.2%), rash (23.8%), elevated triglyceride (21.4%), hypercholesterolemia (21.4%), and oral mucositis (21.4%). The most frequent grade 3 AEs included γ-glutamyl transpeptidase elevation (7.1%), hypertension (4.8%) and hypothyroidism (4.8%). Grade 3 anemia and lymphocytopenia occurred in one and three patients without clinical manifestation, respectively, while no grade 4 or other grade 3 hematological toxicities were observed. No grade 4 AEs were observed except a case of hypokalemia.

**Table 3:** Adverse events occurred in ≥10% of patients overall.

**Events**	**Grade (*****n*****)**			**Total incidence (%)**	**Grade 3 incidence (%)**
	**1**	**2**	**3**		
Diarrhea	11	9	0	47.6	0
Hypertension	14	3	2	45.2	4.8
Hand-foot skin reaction	8	9	1	42.9	2.4
Fatigue	12	4	1	40.5	2.4
Proteinuria	9	6	0	35.7	0
Anorexia	10	3	1	33.3	2.4
Hypothyroidism	6	4	2	28.6	4.8
Hoarseness	10	1	0	26.2	0
Rash	4	6	0	23.8	0
Hypertriglyceridemia	5	3	1	21.4	2.4
Hypercholesterolemia	7	2	0	21.4	0
Oral mucositis	4	5	0	21.4	0
Elevated GGT	5	0	3	19.0	7.1
Hyponatremia	3	4	1	19.0	2.4
Pharyngalgia	5	2	1	19.0	2.4
Low back and leg pain	6	0	0	14.3	0
Elevated TSH	4	1	0	11.9	0
Constipation	5	0	0	11.9	0
Abdominal pain	2	2	1	11.9	2.4
Cough	2	3	0	11.9	0
Vomit	4	1	0	11.9	0

*GGT, γ-Glutamyl transpeptidase; TSH, Thyroid-stimulating hormone*.

Grade 5 AE occurred in one patient, which may be treatment-related. A male patient of 62 years old experienced transient syncope during the second cycle of treatment, and atrial fibrillation was observed in ECG. The patient was treated in the department of cardiology, however, sudden death occurred 4 days later. This patient also experienced grade 2 hypertension during treatment.

## Discussion

In our study, anlotinib demonstrated promising efficacy with confirmed ORR of 16.7%, DCR of 83.3%, and mPFS of 14.0 months in patients with mRCC previously treated with VEGFR-TKI. The mPFS for patients progressed after sunitinib or sorafenib (*n* = 32) was 8.5 months (95% CI 5.6–16.6), indicating the explicit and durable activity of anlotinib in this population.

Although the results could not be compared directly, anlotinib seemed to have numerically better PFS and OS than everolimus which are recommended as the standard second-line treatments. In RECORD-1 study, everolimus prolonged the mPFS of patients failed in previous sunitinib and/or sorafenib from 1.9 to 4.9 months (HR = 0.33, 95% CI 0.25–0.43, *P* < 0.001) compared with placebo and the OS was 14.8 months (30). In another single arm study in Chinese patients with mRCC who were intolerant to or progressed after previous VEGFR-TKIs, everolimus achieved an ORR of 5% and mPFS of 6.9 months (31).

The PFS benefit for anlotinib treatment in this study seemed to be also better than that of axitinib in AXIS study as second-line therapy (mPFS 4.8 months) (28), however, the comparison should be interpreted cautiously. In AXIS study, all the patients enrolled were treated with sunitinib previously, while in our study, sorafenib was used as first-line therapy in 45% (19/42) of the patients. In the western population, sunitinib showed better efficacy than sorafenib in the first-line treatment, while in the eastern population, the advantage seemed to be not obvious (32–34). Cabozantinib is another multi-kinase inhibitor and also showed prominent efficacy in the second-line treatment. In METEOR study, cabozantinib after one or more VEGFR-TKIs showed significantly better efficacy than everolimus (OS 21.4 m vs. 16.5 m, PFS 7.4 m vs. 3.9 m, ORR 17% vs. 3%) (35). Although anlotinb showed the similar mPFS, the comparation of their efficacy will also be interfered by the difference of enrollment.

In another study of anlotinib in mRCC we have published, the efficacy of anlotinib as first-line therapy was comparable with sunitinib (PFS 17.5 m vs. 16.6 m, OS 30.9 m vs. 30.5 m, ORR 30.3% vs. 27.9%) (25). Anyway, these results indicate that anlotinib has prominent clinical activity for mRCC not only in first line therapy, but also in subsequent setting after the failure of previous VEGFR-TKI. Besides, this study further demonstrated the value of sequential anti-angiogenesis therapy in the second-line treatment for mRCC.

In addition to VEGFR and PDGFR, anlotinib strongly inhibits FGFR, which may contribute to its efficacy in second-line therapy. The mechanism for the escape of anti-angiogenesis therapy is complicated and has not yet been clarified completely. The activation of AXL and MET pathways has been found in RCC that are resistant to sunitinib (36). The high potency of cabozantinib in overcoming the resistance to sunitinib may partly result from the inhibition of AXL and MET (37). The up-regulation of PDGF, FGF, angiopoitetin-1, IL-8, Ephrin-A1 and other factors were also found during the treatment with VEGFR2 inhibitors, which may involve in the drug resistance (38). The inhibition of FGFR pathway may enhance the efficacy and partly overcome the resistance of anti-VEGFR therapy. Lenvatinib, a TKI targeting VEGFR, PDGFR, FGFR, RET, and KIT, showed impressing effect after the failure of previous VEGFR-TKI (ORR 27%, DCR 89%, PFS 7.4 m, OS 18.4 m) (39). These results of anlotinib and lenvatinib support the feasibility of simultaneous inhibition of VEGFR, PDGFR, and FGFR as second-line therapy.

In the first-line setting (25), anlotinib showed a better safety profile compared with sunitinib. The incidence of AEs was significantly lower in the anlotinib group, such as hand-foot syndrome (41.1% vs. 65.1%), eyelid edema (2.2% vs. 25.4%), skin yellowing (0% vs. 37.2%), neutropenia (4.4% vs. 46.5%), thrombocytopenia (11.1% vs. 58.1%), anemia (4.4% vs. 34.9%), and taste loss (1.1% vs. 16.7%). Furthermore, the incidence of grade 3 or 4 AEs was also significantly lower in anlotinib group compared with sunitinib group (28.9% vs. 55.8%), especially for thrombocytopenia (0% vs. 11.6%) and neutropenia (0% vs. 9.3%). In this study, anlotinib showed good tolerability again in the second-line setting, as most of the AEs were evaluated as grade 1 or 2. The incidences of most grade 3 or 4 AEs were lower than 5%. The incidence of hematological toxicities such as neutropenia, thrombocytopenia and anemia were lower than 10%. Only three cases of grade 3 lymphocytopenia and one case of grade 3 anemia were observed.

One death induced by arrhythmia occurred during the study, which was judged as treatment-related. In this study, cardiac adverse events were recorded in eight patients, most of which existed before study entry. Only one patient experienced grade 1 QT interval prolongation. In the study of anlotinib as third-line therapy for NSCLC, QT interval prolongation occurred in 25.5% (2.5% were grade 3 or worse) of patients with anlotinib and in 21.9% (1.5% were grade 3 or worse) of patients with placebo (40). The high incidence of adverse events in placebo group may reflect the poor condition of the patients. Generally, the incidence of cardiac toxicity with anlotinib is low. The favorable safety profile indicated the feasibility of anlotinib in combination with other therapy such as checkpoint inhibitors.

Anlotinib was well-tolerable as only five (11.9%) patients needed dose reduction. There was no case of treatment discontinuation. In this study, the scores of FKSI-15 and FKSI-DRS remained stable during anlotinib treatment, suggesting the benefits of anlotinib in delaying the deterioration of disease symptoms and QoL. The consistently high FKSI-15 and FKSI-DRS scores were in accordance with the favorable outcome of these patients.

Our study had limitations. The sample size was relatively small and the comparison of anlotinib with standard second-line regimens needs to be further studied. Besides that, following the development of immunotherapy, the efficacy of combination of anlotinib with checkpoint inhibitors is worth exploring in RCC both as first or second-line.

## Conclusion

This study demonstrated favorable efficacy and safety of anlotinib as second-line treatment for patients with mRCC after a previous VEGFR-TKI, indicating anlotinib may be considered as an option for the second-line treatment of mRCC. Phase 3 trials are required to further elucidate the activity and role of anlotinib in this population.

## Data Availability Statement

The datasets generated for this study are available on request to the corresponding author.

## Ethics Statement

The studies involving human participants were reviewed and approved by National Cancer Center/National Clinical Research Center for Cancer/Cancer Hospital, Chinese Academy of Medical Sciences and Peking Union Medical College. The patients/participants provided their written informed consent to participate in this study.

## Author Contributions

All authors contributed to data collection, data interpretation, and writing of the report. All authors reviewed and approved the submitted article.

## Conflict of Interest

Our study was funded by ChiaTai TianQing Pharmaceutical Group Co., Ltd. The funder had no role in study design, data collection and analysis, decision to publish, or preparation of the manuscript. The declare of authors has been added in the manuscript.
