# Supplementary material for: Anlotinib for Patients With Metastatic Renal Cell Carcinoma Previously Treated With One Vascular Endothelial Growth Factor Receptor-Tyrosine Kinase Inhibitor: A Phase 2 Trial
Source: Front Oncol. 2020 May 7;10:664. doi: 10.3389/fonc.2020.00664 (PMC7221023; doi:10.3389/fonc.2020.00664)
Supplement: Supplementary file 1 [file Table_1.DOCX]

Supplementary table 1. Summary of other adverse events occurred in > 1 patient

| Events | Grade (n) | | | | Total incidence  （%） | Grade 3-4  incidence (%) |
| --- | --- | --- | --- | --- | --- | --- |
|  | 1 | 2 | 3 | 4 |  |  |
| Elevated ALP | 3 | 1 | 0 | 0 | 9.5 | 0 |
| Elevated ALT | 3 | 1 | 0 | 0 | 9.5 | 0 |
| Elevated creatine | 3 | 0 | 1 | 0 | 9.5 | 2.4 |
| Hypophosphatemia | 1 | 2 | 1 | 0 | 9.5 | 2.4 |
| Elevated amylase | 3 | 1 | 0 | 0 | 9.5 | 0 |
| Nausea | 3 | 1 | 0 | 0 | 9.5 | 0 |
| Bloating | 4 | 0 | 0 | 0 | 9.5 | 0 |
| Oral pain | 3 | 1 | 0 | 0 | 9.5 | 0 |
| Anemia | 1 | 2 | 1 | 0 | 9.5 | 2.4 |
| Hair depigmentation | 4 | 0 | 0 | 0 | 9.5 | 0 |
| Headache/dizziness | 3 | 0 | 1 | 0 | 9.5 | 2.4 |
| Palpitation | 3 | 0 | 1 | 0 | 9.5 | 2.4 |
| Chest and back pain | 2 | 2 | 0 | 0 | 9.5 | 0 |
| Lymphocytopenia | 0 | 0 | 3 | 0 | 7.1 | 7.1 |
| Reduced albumin | 1 | 1 | 1 | 0 | 7.1 | 2.4 |
| Leukopenia | 2 | 1 | 0 | 0 | 7.1 | 0 |
| Epistaxis | 3 | 0 | 0 | 0 | 7.1 | 0 |
| Tinnitus | 3 | 0 | 0 | 0 | 7.1 | 0 |
| Aphthous ulcer | 3 | 0 | 0 | 0 | 7.1 | 0 |
| Edema | 2 | 1 | 0 | 0 | 7.1 | 0 |
| Pain | 3 | 0 | 0 | 0 | 7.1 | 0 |
| ECG abnormalities | 3 | 0 | 0 | 0 | 7.1 | 0 |
| Elevated AST | 1 | 1 | 0 | 0 | 4.8 | 0 |
| Elevated DBIL | 1 | 0 | 1 | 0 | 4.8 | 2.4 |
| Elevated hemoglobin | 1 | 1 | 0 | 0 | 4.8 | 0 |
| Elevate LDL | 0 | 1 | 1 | 0 | 4.8 | 2.4 |
| Elevated TBIL | 1 | 1 | 0 | 0 | 4.8 | 0 |
| Fecal occult blood | 1 | 1 | 0 | 0 | 4.8 | 0 |
| Hypokalemia | 1 | 0 | 0 | 1 | 4.8 | 2.4 |
| Hyperuricemia | 2 | 0 | 0 | 0 | 4.8 | 0 |
| Yellow skin | 1 | 1 | 0 | 0 | 4.8 | 0 |
| Asthma | 1 | 1 | 0 | 0 | 4.8 | 0 |
| Stomach pain | 2 | 0 | 0 | 0 | 4.8 | 0 |
| Indigestion | 1 | 1 | 0 | 0 | 4.8 | 0 |
| Hyperglycemia | 2 | 0 | 0 | 0 | 4.8 | 0 |
| Thrombocytopenia | 1 | 1 | 0 | 0 | 4.8 | 0 |
| Neutropenia | 2 | 0 | 0 | 0 | 4.8 | 0 |
| Foot pain | 2 | 0 | 0 | 0 | 4.8 | 0 |

ALT, Alanine transaminase; DBIL, Direct bilirubin; TBIL, Total bilirubin; ALP, Alkaline phosphatase; ECG, Electrocardiograph; LDL, Low density lipoprotein.
